# Immune Subtypes Based on Immune-Related lncRNA: Differential Prognostic Mechanism of Pancreatic Cancer

**DOI:** 10.3389/fcell.2021.698296

**Published:** 2021-07-07

**Authors:** Qiyao Zhang, Zhihui Wang, Xiao Yu, Menggang Zhang, Qingyuan Zheng, Yuting He, Wenzhi Guo

**Affiliations:** ^1^Department of Hepatobiliary and Pancreatic Surgery, The First Affiliated Hospital of Zhengzhou University, Zhengzhou, China; ^2^Key Laboratory of Hepatobiliary and Pancreatic Surgery and Digestive Organ Transplantation of Henan Province, The First Affiliated Hospital of Zhengzhou University, Zhengzhou, China; ^3^Open and Key Laboratory of Hepatobiliary and Pancreatic Surgery and Digestive Organ Transplantation at Henan Universities, Zhengzhou, China; ^4^Henan Key Laboratory of Digestive Organ Transplantation, Zhengzhou, China

**Keywords:** pancreatic cancer, immunotherapy, NMF, prognosis, lncRNA pairs

## Abstract

Pancreatic cancer consists one of tumors with the highest degree of malignancy and the worst prognosis. To date, immunotherapy has become an effective means to improve the prognosis of patients with pancreatic cancer. Long non-coding RNAs (lncRNAs) have also been associated with the immune response. However, the role of immune-related lncRNAs in the immune response of pancreatic cancer remains unclear. In this study, we identified immune-related lncRNA pairs through a new combinatorial algorithm, and then clustered and deeply analyzed the immune characteristics and functional differences between subtypes. Subsequently, the prognostic model of 3 candidate lncRNA pairs was determined by multivariate COX analysis. The results showed significant prognostic differences between the C1 and C2 subtypes, which may be due to the differential infiltration of CTL and NK cells and the activation of tumor-related pathways. The prognostic model of the 3 lncRNA pairs (AC244035.1_vs._AC063926.1, AC066612.1_vs._AC090124.1, and AC244035.1_vs._LINC01885) was established, which exhibits stable and effective prognostic prediction performance. These 3 lncRNA pairs may regulate the anti-tumor effect of immune cells through ion channel pathways. In conclusion, our research demonstrated the panoramic differences in immune characteristics between subtypes and stable prognostic models, and identified new potential targets for immunotherapy.

## Introduction

As one of the most malignant tumors, pancreatic adenocarcinoma (PAAD) is the seventh leading cause of cancer-related deaths, which is responsible more than 430,000 deaths each year worldwide (Bray et al., 2018; Ferlay et al., 2019; Khalaf et al., 2020). Although surgical treatment, radiotherapy, chemotherapy and targeted therapy for PAAD have made significant progress in the past decades, due to the rapid progress of the condition and the limitations of treatment methods, the 5-year survival rate of PAAD patients still does not exceed 5% (David et al., 2009; Von Hoff et al., 2013; Miller et al., 2019). Most PAAD patients still need to rely on chemotherapy and palliative care. However, chemotherapies such as FOLFIRINOX can increase the patients’ median survival time only by 2–4 months and have obvious side effects (Vaccaro et al., 2011). Therefore, there is an urgent need to further explore the mechanism of occurrence and development of pancreatic cancer, as well as to find novel therapeutic targets to improve the prognosis of PAAD patients.

In recent years, immunotherapy has made tremendous breakthroughs and seems to have become a new hot topic in cancer treatment (Mellman et al., 2011; Chen et al., 2017). Immune checkpoint inhibitors (ICIs) have been used in a variety of cancers including pancreatic cancer (Long et al., 2017; Wu et al., 2019). ICIs can restore the anti-tumor response of the immune system and prevent tumors from evading immune surveillance through immune checkpoint signaling pathways (Kythreotou et al., 2018; Yi et al., 2018). However, immunotherapy improves only some PAAD patients’ condition (Glatzer et al., 2020). Therefore, exploring the immune characteristics of pancreatic cancer and finding new immunotherapy targets are of great significance for improved immunotherapy effects for patients.

Long non-coding RNAs (lncRNAs) are RNAs with a length longer than 200 nucleotides, which are generally considered not translated directly into proteins. Instead, as indicated by a large number of studies in recent years, lncRNAs regulate translation efficiency by binding to mRNAs and exert their biological functions in this manner (Mercer et al., 2009; Castellanos-Rubio and Ghosh, 2019). Evidence shows that lncRNAs are potential immune regulators, deeply involved in cellular immune and inflammatory processes (Geng and Tan, 2016; Chen J. et al., 2019). For example, as a pseudogene of Rps15a-ps4, lncRNA Lethe can block NF-κB-DNA binding, thereby promoting the anti-inflammatory effect of dexamethasone (Rapicavoli et al., 2013). However, the mechanism of how immune-related lncRNAs affect the prognosis of PAAD patients is still not fully understood.

In this study, we identified immune-related lncRNA pairs by combining the lncRNA expression profile data of PAAD patients with the immune gene library, and clustered two molecular subtypes based on this pairing. We then comprehensively analyzed the differences in prognosis, immune characteristics, gene mutations and potential functions between subtypes. Finally, univariate and multivariate cox analyses were performed to construct a prognostic model based on 3 selected lncRNA pairs. After a variety of verifications, the model was proven to have stable and independent prognostic prediction performance.

## Materials and Methods

### Data Source and Preprocessing

The most up-to-date expression profile data and clinical follow-up information of PAAD patients were downloaded from the TCGA database^1^ on March 17, 2021. Subsequently, we processed the RNA-Seq data of TCGA-PAAD according to the following steps: (1) Remove samples without clinical follow-up information; (2) Remove samples without survival time; (3) Exclude samples without survival status; (4) Convert ensemble to gene symbol; (5) Take genes with multiple Gene Symbols as the median value of their expression. The TCGA-PAAD cohort after data preprocessing contained a total of 176 samples. The expression profile data and follow-up information of the ICGA-PACA-CA cohort (167 samples in total) were downloaded from the ICGC database^2^.

### Identification and Pairing of IRGs and lncRNAs

The immune-related gene (IRG) set was downloaded from the ImmPort database^3^, which contains the comprehensive location information and functional attributes of immune-related genes.

The expression profile of TCGA-PAAD was divided into mRNAs and lncRNAs based on the latest version expression profile annotation file downloaded from the GENCODE website^4^. We calculated the co-expression Pearson correlation coefficient and *p*-value among each IRG and lncRNA. According to the threshold of Cor > 0.8 and *P* < 0.01, a total of 1,289 lncRNA-IRG pairs were identified, including 466 lncRNAs and 228 IRGs (Supplementary File 1). Next, 466 immune-related lncRNAs were paired in a cycle. In order to eliminate the huge difference among the expression of lncRNAs, we processed the data as follows: we defined C as the expression of the lncRNA pair (lncRNA A and lncRNA B). If the expression level of lncRNA A was higher than lncRNA B, then C was defined as 1; otherwise, C was defined as 0. Based on this technique, we constructed a matrix containing values of 0 and 1. Next, lncRNA pairs with C = 1 accounting for 30∼70% of all lncRNA pairs were retained (Hong et al., 2020).

### Identification of Immune-Related Molecular Classes Based on NMF

For the lncRNA pairs obtained by the above processing method, we used the coxph function in R to perform univariate cox analysis, and thus obtained 217 lncRNA pairs related to the prognosis of PAAD (*P* < 0.001). Subsequently, the non-negative matrix factorization (NMF) algorithm was used to cluster the PAAD samples. The method was set as the standard called “brunet” that performs 100 iterations. The number of clusters k was set from 2 to 10, the average contour width of the shared member matrix was determined by the R package NMF, and the minimum member of each sub-category was set to 10.

### Immune Characteristics and Tumor Mutation Burden (TMB) Analysis

The characteristics of the 22 immune cells in each sample between subtypes were determined based on the R package CIBERSORT (Chen et al., 2018). The mutation dataset of the TCGA-PAAD cohort was downloaded from the TCGA database and processed by Mutect2 software to analyze the tumor mutation burden (TMB) of each sample.

### Construction of Prognostic Risk Model Based on Immune-Related lncRNA Pairs

We used the Fisher’s exact test to calculate the differences between the subtypes of each lncRNA pair, and then obtained the adjusted FDR values by the BH method. With FDR <0.0001 as the threshold, we identified a total of 390 differential lncRNA pairs (Supplementary File 2). Subsequently, we divided the 176 samples of the TCGA-PAAD queue into a training set and a validation set. In order to ensure the stability of subsequent modeling, all samples were randomly grouped at 1:1 for 100 times with replacement. We chose the best grouping based on the criteria of no significant difference in age distribution, gender, follow-up time, and the proportion of deaths between the two groups. A total of 88 samples were included the training set and 88 samples in the validation set. As shown in Table 1, there was no significant difference between the groups (*P* > 0.05).

**TABLE 1:** Differences in clinical characteristics between training set and validation set.

Clinical Features	TCGA-train	TCGA-test	*P*
**Event**			
Alive	42	42	1
Dead	46	46	
**Stage**			
I	12	9	0.2715
II	74	71	
III	1	2	
IV	0	4	
X	1	2	
**Grade**			
G1	16	14	0.3309
G2	50	44	
G3	19	29	
G4	2	0	
GX	1	1	
**Age**			
≤65	46	47	1
>65	42	41	
**T Stage**			
T1	6	1	0.3342
T2	10	14	
T3	70	70	
T4	1	2	
TX	1	1	
**N Stage**			
N0	26	23	0.3709
N1	61	61	
NX	1	4	
**M Stage**			
M0	40	39	0.1281
M1	0	4	
MX	48	45	
**Gender**			
Female	39	41	0.8797
Male	49	47	
**Alcohol**			
NO	32	32	0.4739
YES	48	52	
Unknown	8	4	
**Radiation_therapy**			
NO	53	48	0.6802
YES	14	18	
Unknown	21	22	
**Chemotherapy**			
NO	30	30	1
YES	58	58	

The multivariate Cox proportional hazard regression model was carried out for the different lncRNA pairs between subtypes using the R survival package, coxph function based on training set. The significance level of *P* < 0.05 was set as the threshold for filtering. Finally, we performed a multivariate COX analysis on the significantly different lncRNAs to obtain the risk coefficients of lncRNA pairs.

### Functional Enrichment Analysis

**FIGURE 1:**
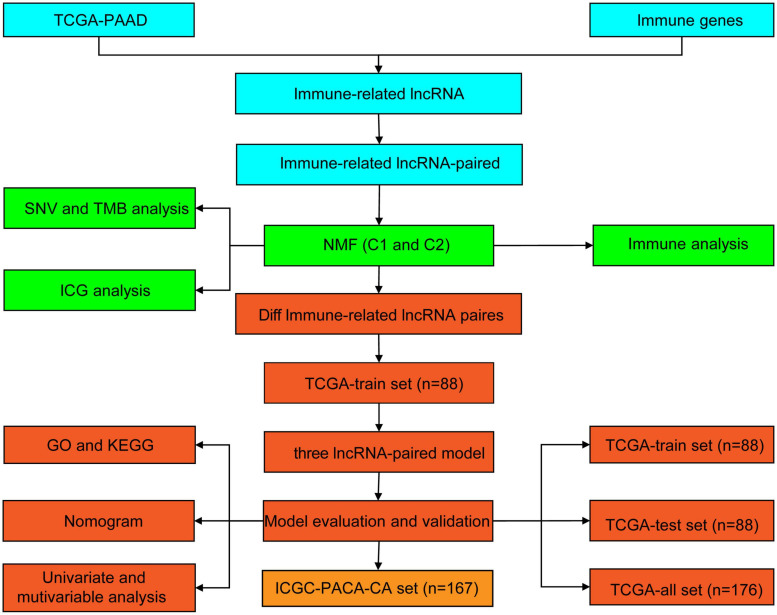
Flowchart of the study.

Gene Ontology (GO) is a structured method of gene product annotation, which consists of three parts: biological process (BP), cell component (CC), and molecular function (MF). The Kyoto Encyclopedia of Genes and Genomes (KEGG) is an open gene set pathway enrichment database. In this study, GO and KEGG were performed to further understand the functional differences between subtypes using the R package WebGestaltR (v0.4.2) (Wang et al., 2017). The GO terms and KEGG pathways with *P* < 0.05 were considered to be significantly different. All analytical processes are described in Figure 1.

## Results

### Molecular Typing of PAAD Based on Immune-Related lncRNA Pairs

**FIGURE 2:**
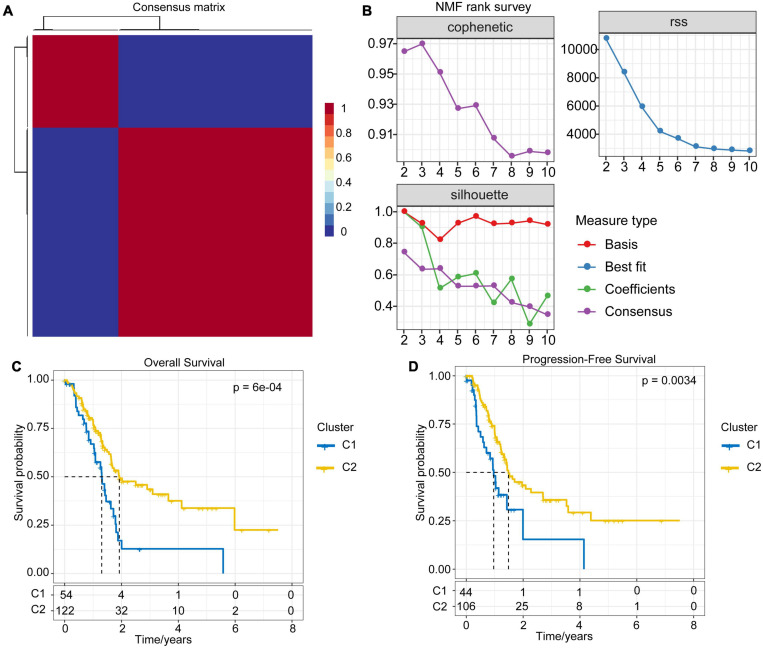
NMF algorithm clustering and prognostic differences between subtypes. **(A)** Consensus map of NMF clustering. **(B)** The cophenetic, RSS and dispersion distributions with rank = 2–10; combining these indicators results in the optimal number of clusters of 2. **(C)** OS time prognostic survival curve of the PAAD molecular subtype. **(D)** PFS time prognostic survival curve of the PAAD molecular subtype.

The immune-related lncRNA pairs were identified through the cyclic pairing of immune lncRNAs. Subsequently, we performed univariate COX analysis of these lncRNA pairs using the coxph function in R. A total of 217 prognostic-related (*P* < 0.001) lncRNA pairs for PAAD were obtained (Supplementary File 3). Next, we clustered PAAD samples by non-negative matrix clustering algorithm (NMF) based on the prognostic-related lncRNA pairs (Supplementary Figure 1). According to the indicators, such as cophenetic, dispersion and silhouette, we determined the optimal number of clusters as 2 (Figures 2A,B). Accordingly, we divided the samples of the TCGA-PAAD cohort into C1 and C2 subtypes. The further survival analysis between subtypes showed that there were significant differences between them either in terms of overall survival time or progression-free survival (PFS) time. The prognosis of the C1 subtype was much worse than that of the C2 subtype (Figures 2C,D).

### Differences of TMB and Common Gene Mutations Between Immune Subtypes

**FIGURE 3:**
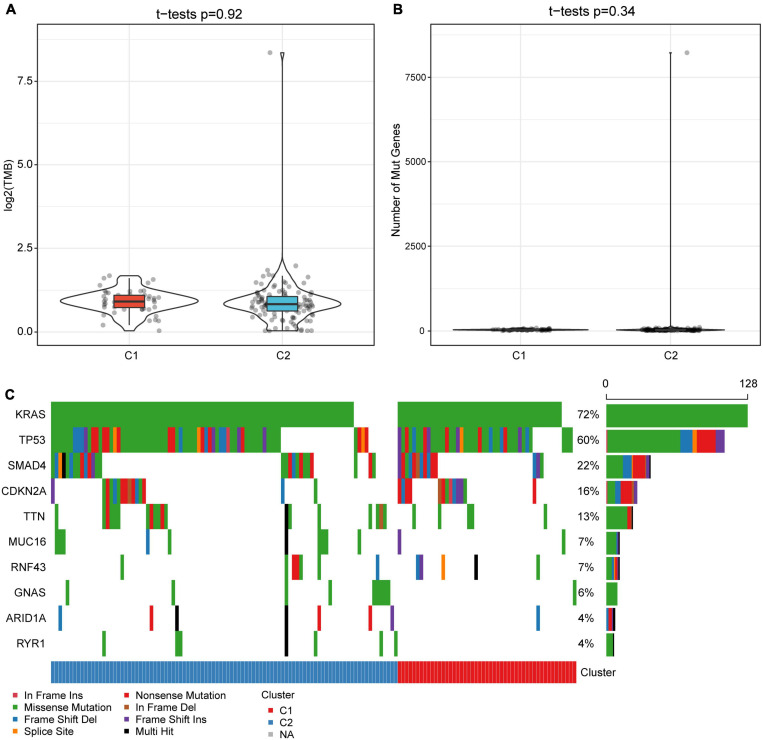
Tumor mutation burden and gene mutation characteristics among molecular subtypes. **(A)** Differences in the distribution of TMB between subtypes. **(B)** The distribution difference of the number of gene mutations between subtypes. **(C)** Mutation characteristics of top 10 genes in two subtype samples. The rank sum test is used to determine the *p*-value.

The gene mutation dataset of TCGA-PAAD was downloaded to understand the differences in TMB and gene mutations between subtypes. Results showed that the TMB of the C1 subtype was slightly higher than that of the C2 subtype, although no significant statistical difference was detected (Figure 3A). Meanwhile, we also assessed the differences in the number of mutant genes between the samples (Figure 3B). There was no difference in the number of mutant genes between the C1 and C2 subtypes. In addition, we showed the mutation characteristics of the top 10 genes with the most frequent mutations in each subtype (Figure 3C). Consistently with previous reports, most of the mutations detected in the two subtypes were missense mutations (Zhang et al., 2020). Specifically, only the mutation rate of TP53 in the C1 subtype was significantly higher than that of the C2 subtype (*P* = 0.036).

### Differences in Immune Characteristics and Pathway Characteristics Between Subtypes

**FIGURE 4:**
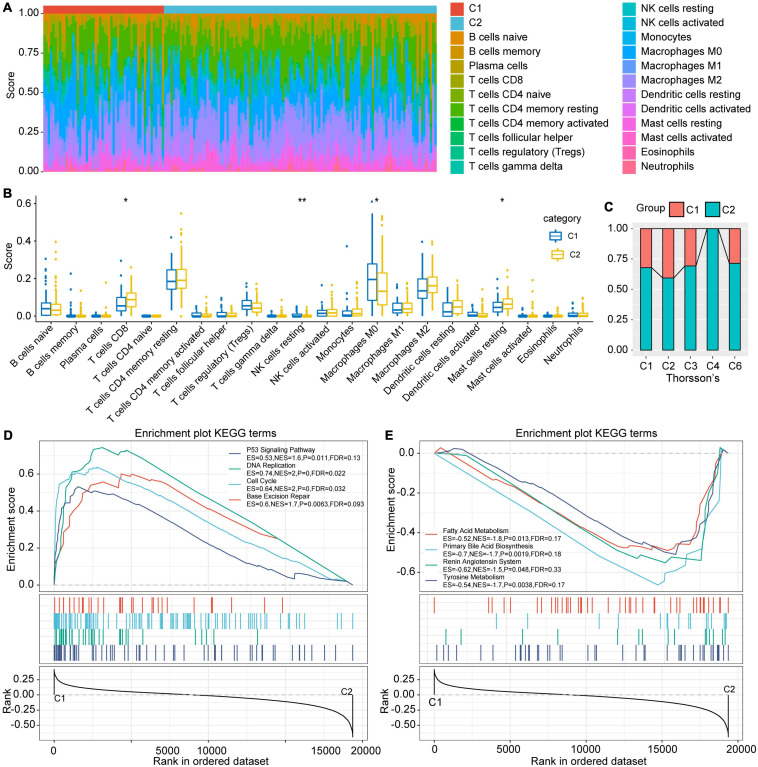
Differences in immune cell characteristics between subtypes and GSEA. **(A)** Ratio of 22 immune cell components of the 2 subtype samples. **(B)** Differences in scores of 22 immune cells in samples between subtypes. **(C)** Intersection of C1 and C2 with the previous pan-cancer immune molecular subtypes. **(D)** The KEGG pathways enriched in C1 subtypes are mainly tumor-related pathways. **(E)** The KEGG pathways enriched in the C2 subtype are mainly metabolic related pathways, *indicates less than 0.05; **indicates less than 0.01.

In order to explore the immune characteristics of the C1 and C2 subtypes, we evaluated the immune cell score of each sample with CIBERSORT (Figure 4A). The immune cell scores obtained were different both within and between groups. After statistical testing, we established that the T cell CD8 and Mast cell resting scores in C1 subtype were significantly lower, while the NK cell resting and macrophage M2 cell scores were significantly higher than those in C2 subtypes (Figure 4B). Meanwhile, activated NK cells had a higher score in the C2 subtype, although the difference was not statistically significant. The above results suggest that the poor prognosis of the C1 subtype may be partly due to the inactivation of CTLs and NK cells in the C1 subtype, which causes the immune escape of the tumor. The score of non-polarized M0 macrophages in the C1 subtype was significantly higher than that in C2, which leads to the assumption that the activation of macrophages in C1 subtype was inhibited. Paradoxically, there was no significant difference between M1 and M2, which finding requires further exploration.

### Comparison Between TCGA Molecular Subtypes and Existing Immune Subtypes

Thorsson et al. (2018) conducted an extensive tumor immunophenotyping test using more than 10,000 samples of 33 cancers in TCGA. A total of 6 subtypes were identified: wound healing (C1), IFN-gamma dominant (C2), inflammatory (C3), lymphocyte depleted (C4), immunologically quiet (C5), and TGF-beta dominant (C6). Among them, C1 and C2 subtypes correspond to poor prognosis, while C3, C4, and C6 have tumor suppressor effects (Thorsson et al., 2018). By comparing Thorsson and colleague’s immune subtypes with those established in our study, results showed that our C1 subtype mostly corresponded to Thorsson’s C1 and C2 subtypes, while our C2 subtype had a higher ratio of Thorsson’s C3, C4, and C6 (Figure 4C). This also illustrates the stability of the subtypes identified herein.

### Gene Set Enrichment Analysis (GSEA) Among Subtypes

The process of GSEA was performed to explore the significantly enriched pathways in each subtype. *P* < 0.05 and FDR < 0.25 were set as thresholds to select the enrichment pathways. The results showed that multiple tumor-related pathways, including P53 signaling pathway, DNA replication, Cell cycle, and Base excision repair were enriched in the C1 subtype, while the metabolism-related pathways such as Fatty acid metabolism, Primary bile acid biosynthesis, Renin angiotensin system and Tyrosine metabolism were enriched in C2 (Figures 4D,E). This implies that the poor prognosis of C1 may be due to the further activation of tumor-related pathways and the inhibition of normal metabolism.

### Differences in Intrinsic Immune Escape Characteristics Between Subtypes

**FIGURE 5:**
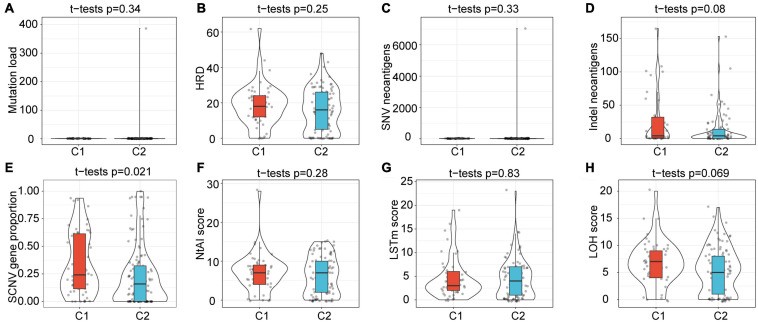
Differences in factors related to innate immune escape between subtypes. **(A–H)** Differences among subtypes in Mutation load, HRD, SNV neoantigens, Indel neoantigens, SCNV gene proportion, Ntal score, LSTm score and LOH score. Among them, only the SCNV gene proportion shows significant difference between subtypes.

The intrinsic immune escape of tumors suggests that tumor cells directly mediate their own immune escape, which leads to tumor progression. The study of Schreiber et al. (2011) proved that tumor immunogenicity and the expression of immune checkpoint molecules were two aspects of intrinsic immune escape. Herein, to explore the differences in the intrinsic immune escape characteristics between the subtypes, we compared the potential factors that affect tumor immunogenicity, including mutation load, homologous recombination deficient (HRD), neoantigen load and chromosomal instability levels, as well as other factors (Figure 5). The results showed that most of the factors affecting tumor immunogenicity did not differ between the subtypes, while the SCNV gene proportion in the C1 subtype was significantly higher than that in C2.

### Prognostic Risk Model Based on Immune-Related lncRNA Pairs

In order to further explore the key differential lncRNA pairs affecting the prognosis between subtypes and their prognostic prediction ability for PAAD patients, we performed univariate COX proportional hazard regression on differentially expressed lncRNA pairs in the training set, where *P* < 0.05 was considered as a significant difference. A total of three prognostic-related differential lncRNA pairs were identified: AC244035.1_vs._AC063926.1, AC066612.1_vs._AC090124.1, and AC244035.1_vs._LINC01885. Subsequently, multivariate COX analysis was performed for these 3 prognostic-related lncRNA pairs to obtain the risk coefficient of each lncRNA pair. Based on these coefficients, the RiskScore formula was acquired as follows:

RiskScore=(−0.193A*C244035.1_vs._AC063926.1)      +(−0.445*AC066612.1_vs._AC090124.1)+(−0.504*AC244035.1_vs._LINC01885)

All of these 3 lncRNA pairs were established as protective factors of PAAD.

**FIGURE 6:**
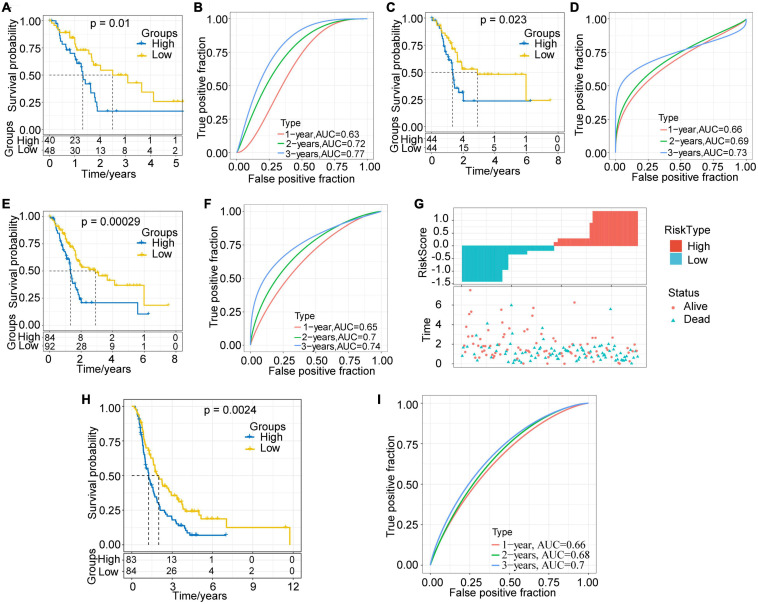
Evaluation and validation of prognostic models. **(A)** KM survival curve distribution of the high-risk group and the low-risk group in the training set. **(B)** ROC curve of the prognostic model in the training set. **(C)** KM survival curve distribution of the high-risk group and the low-risk group in the validation set. **(D)** ROC curve of the prognostic model in the validation set. **(E)** KM survival curve distribution of the high-risk group and the low-risk group in all samples of the TCGA-PAAD cohort. **(F)** ROC curve of the prognostic model of all samples. **(G)** Correlations among RiskScore, survival time and survival status of the TCGA-PAAD cohort; RiskScore is arranged from low to high. **(H)** KM survival curve distribution of the high-risk group and the low-risk group in the ICGC-PACA-CA cohort. **(I)** ROC curve of the prognostic model in the ICGC-PACA-CA cohort.

The RiskScore of each sample in the training set was calculated, and the Z-score was normalized. Samples with a RiskScore > 0 were classified as high-risk groups, otherwise they were categorized as low-risk groups. Among them, 40 samples were associated with high-risk groups, and 48 samples were classified as low-risk groups. The survival analysis showed that, as expected, the prognosis for the high-risk group was significantly worse than that for the low-risk group (*P* = 0.01, Figure 6A). We further performed receiver operating curve analysis using the R software package timeROC. The prognostic prediction power (AUC) of this prognostic model was 0.63 (1 year), 0.72 (2 years), and 0.77 (3 years), respectively (Figure 6B). Therefore, our model showed a relatively good long-term survival prediction performance.

### Validation of Robustness of the Risk Model in the Internal and External Validation Sets

With the aim to verify the robustness of the model, we calculated the RiskScore of each sample in the validation set and all TCGA-PAAD samples with the same model and coefficients as in the training set. In the validation set, the prognosis for the high-risk group proved much worse than that for the low-risk group (*p* = 0.023, Figure 6C). The results of ROC analysis also showed that the AUC of this prognostic model were 0.66 (1 year), 0.69 (2 years), and 0.73 (3 years), respectively (Figure 6D). In the TCGA-PAAD cohort, consistently with our expectations, the prognosis of different risk groups showed extremely significant difference (*P* = 0.00029, Figure 6E). Its 1–, 2–, and 3-year AUC values were established as 0.65, 0.7, and 0.74, respectively (Figure 6F).

In addition, we plotted the distribution of RiskScore and the survival status of all samples (Figure 6G). The results showed that the RiskScore was significantly correlated with the patient survival status. It could be seen that, as the RiskScore increased, the number of alive patients and the survival time significantly reduced. The above results imply that the prognostic model based on the identified 3 lncRNA pairs has a high and stable predictive power for the long-term survival rate of PAAD patients. Furthermore, we adopted the ICGC-PACA-CA cohort to verify the effectiveness of the model. The results showed that the prognosis of the high-risk group was significantly worse than that of the low-risk group (*p* = 0.0024, Figure 6H). The 1–, 2–, and 3-year AUC of the prediction model were 0.66, 0.68, and 0.7 (Figure 6I), respectively, showing the cross-platform effectiveness of the model.

### Differences in the RiskScores of Various Clinical Characteristics

**FIGURE 7:**
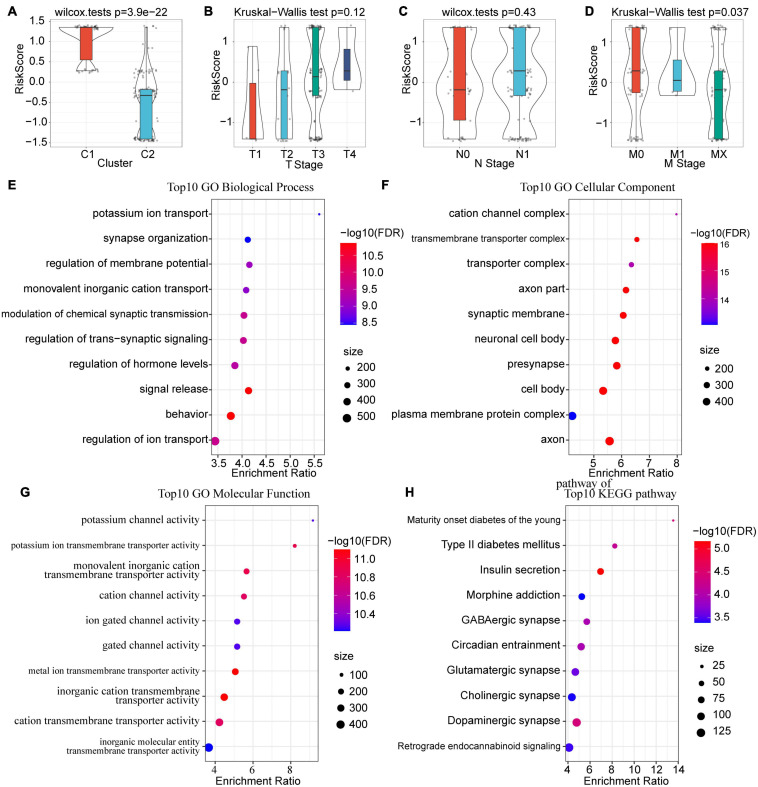
Correlations among risk score, clinical characteristics and functional enrichment analysis. **(A)** Differences in risk scores between subtypes. **(B)** Differences in RiskScores for T stage. **(C)** Differences in RiskScores for N stage. **(D)** Differences in RiskScores for M stage. **(E)** Top 10 BP enrichment terms of lncRNA-related mRNAs. **(F)** Top 10 CC enrichment terms of lncRNA-related mRNAs. **(G)** Top 10 MF enrichment terms of lncRNA-related mRNAs. GO terms were mainly enriched in related pathways of ion channels. **(H)** Top 10 KEGG pathways of lncRNA-related mRNAs.

Furthermore, we compared the differences in risk scores of clinical features, including TNM stage, grade, molecular subtype, etc. Results indicated that different clusters and M stages have significant differences in their risk scores (*P* < 0.05). The risk score of C1 was much higher than that of C2, which corresponds to the poorer prognosis of C1 (Figure 7A). Meanwhile, a trend was observed that the risk score increases with the advancement of T stage and N stage, although the difference was not statistically significant (Figures 7B,C). Unexpectedly, however, the risk score of MX stage was lower than that of M1 and M0 (Figure 7D). This means that the invasion ability of samples with high RiskScore was decreased. Moreover, we compared the differences in risk scores for age, gender, and treatment, and none of them were statistically significant (Supplementary Figure 2).

### Identification and Functional Analysis Genes Related to the 3 lncRNA Pairs

Considering the fact that lncRNAs usually binds with mRNA and proteins to perform biological functions, we calculated the Spearman correlation coefficient and their significance between the 3 prognostic-related lncRNA pairs and mRNA. After filtering with a threshold of Corr > 0.4 and *P* < 0.05, a total of 553 genes were identified. Subsequently, GO and KEGG analyses were performed to explore the potential functions of these genes. For biological functions (BP), 216 items were identified with significant differences (FDR < 0.05), which are mainly related to ion transport and the regulation of transmembrane signals (Figure 7E); for molecular functions (MF), there were 62 items with significant differences (FDR < 0.05), mainly concentrated on the cation channel complex and transmembrane transport complex (Figure 7F); for cell components (CC), 93 entries showed significant differences (FDR < 0.05), and these functions were mainly associated with ion and protein transport channels (Figure 7G). For KEGG, pathways such as Maturity onset diabetes of the young, Type II diabetes mellitus, Insulin secretion, Morphine addiction, GABAergic synapse, and Circadian entrainment were enriched (Figure 7H).

### Univariate and Multivariate Analysis of RiskScore

**FIGURE 8:**
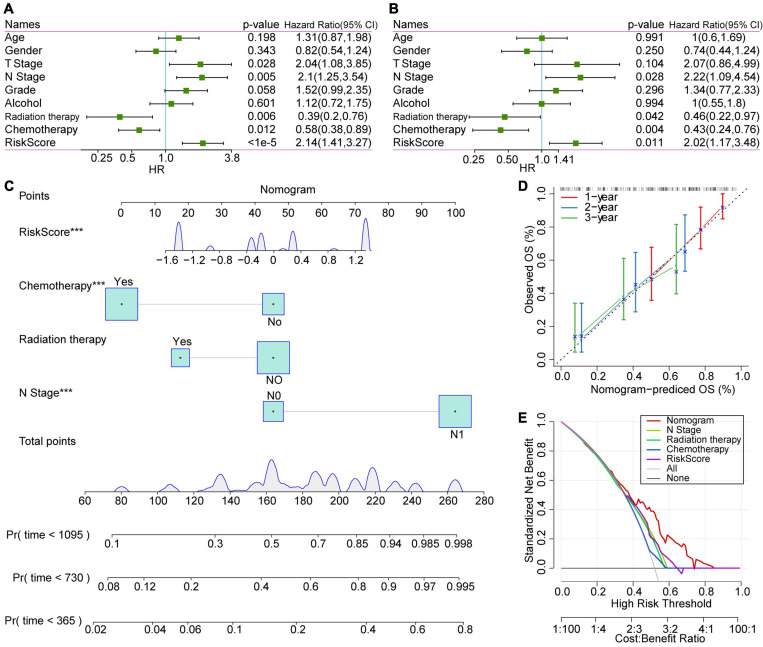
COX analysis of RiskScores, clinical characteristics and nomogram. **(A)** Univariate analysis results of clinical features and RiskScore. **(B)** Multivariate analysis results of clinical features and RiskScore. **(C)** Nomogram based on clinical characteristics and RiskScore. **(D)** Nomogram survival rate correction chart. **(E)** Decision curve analysis (DCA) diagrams of N stage, radiation therapy, chemotherapy, RiskScore, and nomogram.

Aiming to verify the stability and independence of the RiskScore determined in clinical applications, we performed a univariate COX regression analysis on the TCGA-PAAD samples. The results showed that T Stage, N Stage, and RiskScore were negatively correlated with patient prognosis (HR > 1, *P* < 0.05), while radiation therapy and chemotherapy were positively correlated with patient prognosis (HR < 1, *P* < 0.05, Figure 8A). Among them, the RiskScore had the highest Hazard Ratio (HR = 2.14), which also proved that it was highly effective in predicting the prognosis of patients. Meanwhile, the results of multivariate analysis showed that the HR of RiskScore we determined was 2.02 (*P* = 0.011), which still had a strong power in predicting the prognosis of PAAD patients (Figure 8B). The above results prove that our prognostic model based on the selected 3 lncRNA pairs has strong independent predictive power.

### Construction of Nomogram of Clinical Characteristics and DCA Curve

A nomogram is a figure that visually and effectively displays the results of a risk model. It uses the length of the straight line to indicate the degree of influence of different variables and the different values of these variables on the outcome. We constructed the nomogram based on the clinical features with significant statistical significance in the multivariate COX regression analysis. The results of the nomogram showed that the RiskScore had the greatest impact on the prognostic outcome of patients and was relatively stable (Figure 8C), indicating that the risk model based on our lncRNA pairs can stably predict the prognosis of patients. Furthermore, we verified the performance of nomogram data for predicting patient prognosis. We observed that the nomogram-predicted OS data fit the observed OS well with respect to 1–, 2–, and 3-year survival rates, which proves that this method presents excellent performance (Figure 8D).

Moreover, decision curve analysis (DCA) was performed to evaluate the net benefits of different characteristics for patients (Figure 8E). Subsequent results showed that decisions based on nomogram data have the highest net benefits. Meanwhile, the decision based on the RiskScore had a good impact on the patients’ net benefits, and its net income was higher than the N stage in most cases.

## Discussion

It has been established that PAAD can mediate a unique immune microenvironment and cause immune escape through a variety of mechanisms, such as through tumor “hijacking” immune checkpoints to suppress the immune system’s anti-tumor response (Collisson et al., 2019; Liu et al., 2020). ICIs effectively prevent this process (Ribas and Wolchok, 2018). Notwithstanding, there are still patients who do not respond to ICIs, and even present negative outcomes (Macherla et al., 2018). However, lncRNAs, which are closely related to immunity, seem to be seldom subject to research in this field. Therefore, exploring the mechanism of immune-related lncRNA in PAAD patients may bring unexpected gains.

In this study, we used the lncRNA expression profile of the TCGA-PAAD cohort to identify immune-related lncRNAs combined with the immune dataset. In terms of the accuracy of the cancer diagnosis model, the combination of two biomarkers is better than a single gene, therefore we screened immune-related lncRNA pairs through iteration and pairing, and constructed a new expression matrix based on the relative differences in the expression levels within the combination to eliminate huge differences in expression (Hong et al., 2020; Lv et al., 2020). Based on the expression matrix of the immune-related lncRNA pairs, we determined 2 molecular subtypes using the NMF algorithm. It was found that C1 and C2 had significant differences in overall survival and PFS. Subsequently, the molecular level differences between subtypes were further explored. The TMB proved to have no significant difference between the groups, and the mutation rate of TP53 in the CI subtype was significantly higher than that in the C2 subtype. TP53 is a thoroughly studied tumor suppressor gene, whose translation product P53 can activate target genes to resist cell stress and mediate cell growth arrest and apoptosis (Levy et al., 1993; Kanda et al., 2013; Ormanns et al., 2014). Elevated TP53 mutations may cause the loss of P53 protein function and lead to further uncontrollable tumor proliferation, which may be partly the reason for the poor prognosis of C1.

It was further established by the analysis of the immune characteristics between subtypes that the infiltration of CTLs in the C1 subtype was reduced, while the proportion of resting NK cells increased, suggesting that activated NK cells were reduced and the anti-tumor effect of non-specific immunity was suppressed. Zhang et al. (2017) reported that the immunosuppressive state of a tumor microenvironment can cause NK cell dysfunction, which corresponds to poor prognostic outcomes. Previous evidence has indicated that M1 phase macrophages have anti-tumor effects, whereas M2 phase macrophages have tumor-promoting effects (Chen Y. et al., 2019; Vitale et al., 2019). Interestingly, our results showed that there were more non-polarized M0-phase macrophages in C1 subtypes than in C2 subtypes. Meanwhile, there was no significant difference in M1 and M2 phase macrophages between subtypes. Based on this finding, it can be inferred that the C1 subtype may inhibit the polarization of M0 macrophages through a certain mechanism to exert an effect, which speculation requires more rigorous experiments to validate. In addition, GSEA results suggest that some tumor-related pathways, such as P53 signaling pathway, DNA replication, cell cycle, and base excision repair, are enriched in the C1 subtype, while fatty acid metabolism, primary bile acid biosynthesis, renin, angiotensin, methionine and tyrosine metabolism are enriched in the C2 subtype, indicating that the poor prognosis of C1 subtype is related to the activation of tumor-related pathways and the inhibition of normal metabolism.

Subsequently, based on univariate and multivariate COX analysis, we constructed a prognostic model of the selected 3 immune-related lncRNA pairs (AC244035.1_vs._AC063926.1, AC066612.1_vs._AC090124.1, and AC244035.1_vs._LINC01885). After rigorous verification, the model proved to have a stable and independent prognostic prediction performance, and its long-term prognosis AUC reached 0.77. The RiskScore was found to be significantly related to molecular subtype and M stage. The functional enrichment analysis of mRNAs related to these 3 lncRNA pairs revealed that they are mainly involved in ion transport pathways. There is evidence that K^+^ channels (Kv1.3 channels) accumulate specifically in the immune synapse between CTL and tumor cells to regulate the cell killing effect of CTL and NK cells (Panyi et al., 2004; Hu et al., 2013). Blocking this channel can enhance the tumor killing effect. The Ca^2+^-activated K^+^ channels play a key role in the development and metastasis of tumors (Khaitan et al., 2009; Hanahan and Weinberg, 2011; Schwab et al., 2012). We have reason to believe that the proposed three immune-related lncRNA pairs may regulate the anti-tumor effect of immune cells and the process of tumor invasion through ion channel-related pathways, therefore may become new targets for tumor treatment.

## Conclusion

In this study, we identified new PAAD molecular subtypes with significant prognostic differences based on immune-related lncRNA pairs. The detected prognostic differences between subtypes may be due to the differential infiltration of CTL and NK cells, and the activation of tumor-related pathways. In addition, the prognostic model based on the 3 identified immune-related lncRNA pair signatures was proved to have an effective and stable prognostic predictive effect on PAAD. The proposed 3 lncRNA pairs may participate in the anti-tumor effect of immune cells and tumor migration through ion channel pathways, and are expected to become new tumor treatment targets.

## Data Availability Statement

The datasets presented in this study can be found in online repositories. The names of the repository/repositories and accession number(s) can be found in the article/Supplementary Material.

## Author Contributions

YH and WG designed the study. QZa and QZe drafted the manuscript. QZa and ZW analyzed the data. XY and MZ revised the manuscript. All authors read and approved the final manuscript.

## Conflict of Interest

The authors declare that the research was conducted in the absence of any commercial or financial relationships that could be construed as a potential conflict of interest.

## Footnotes

1 https://portal.gdc.cancer.gov/

2 https://daco.icgc.org/

3 http://www.immport.org

4 https://www.gencodegenes.org

## Supplementary Material

Supplementary Figure 1 Consensus map of NMF clustering when *K* = 2–10.

Supplementary Figure 2 Correlation between risk score and clinical characteristics.
