# Supplementary figures and images for: Immune Subtypes Based on Immune-Related lncRNA: Differential Prognostic Mechanism of Pancreatic Cancer
Source: Front Cell Dev Biol. 2021 Jul 7;9:698296. doi: 10.3389/fcell.2021.698296 (PMC8292792; doi:10.3389/fcell.2021.698296)

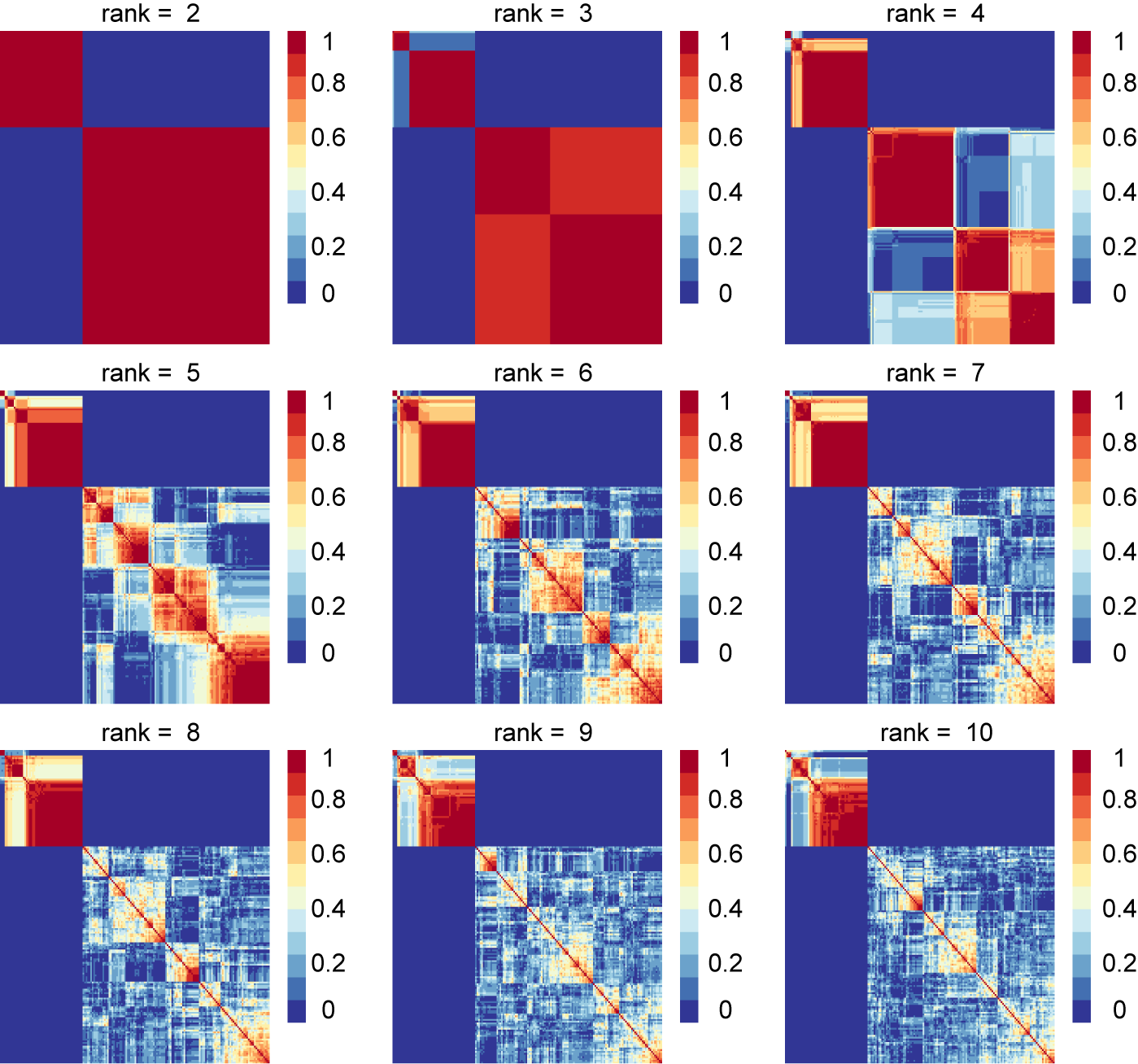

Supplement: Supplementary Figure 1 — Consensus map of NMF clustering when K = 2–10. [file Image_1.TIF]

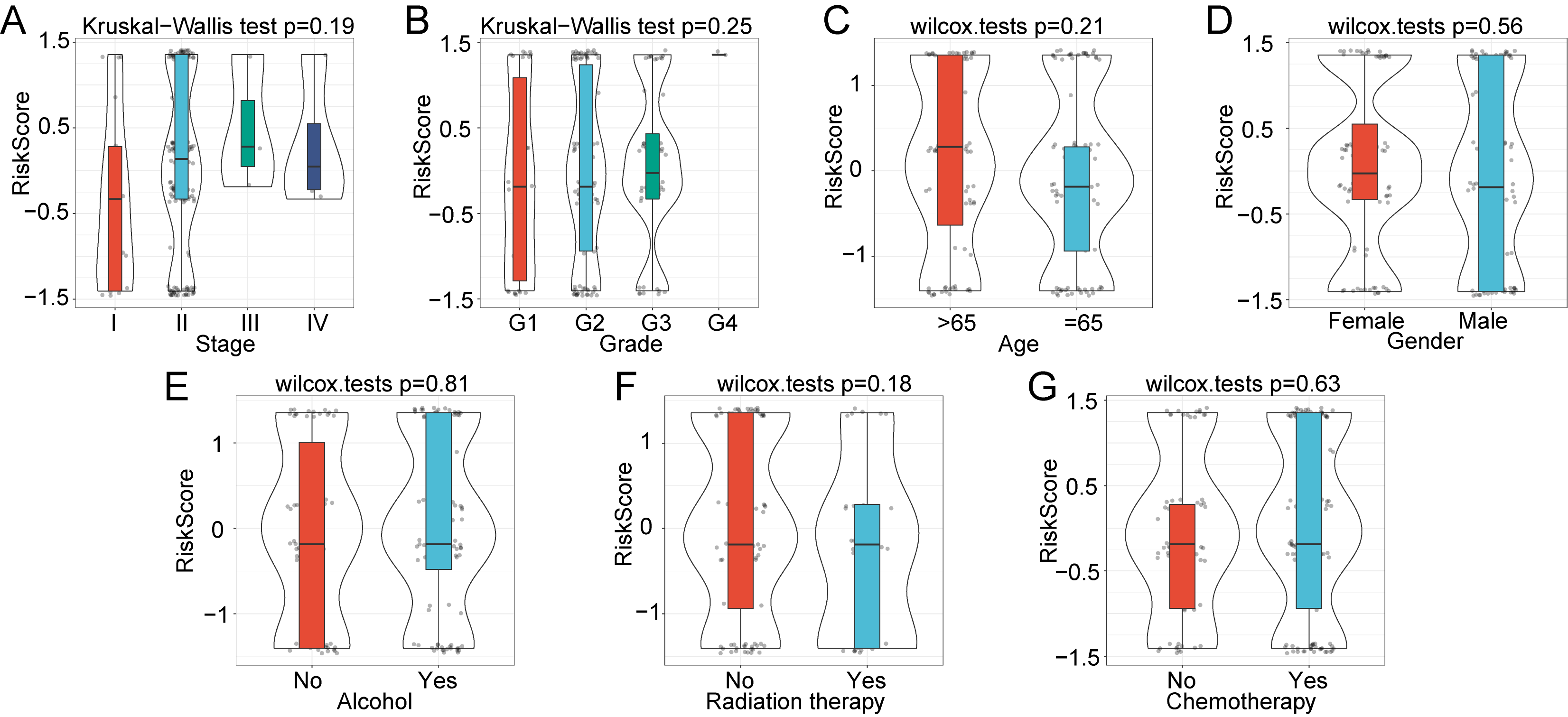

Supplement: Supplementary Figure 2 — Correlation between risk score and clinical characteristics. [file Image_2.TIF]
