# Supplementary material for: Immune Subtypes Based on Immune-Related lncRNA: Differential Prognostic Mechanism of Pancreatic Cancer
Source: Front Cell Dev Biol. 2021 Jul 7;9:698296. doi: 10.3389/fcell.2021.698296 (PMC8292792; doi:10.3389/fcell.2021.698296)
Supplement: Supplementary file 6 [file Data_Sheet_4.ZIP › Supplementary/Statistics of clinical information of TCGA-PAAD patients.docx]

**Table 1: Statistics of clinical information of TCGA-PAAD patients**

| **Clinical Features** | **TCGA-PAAD** |
| --- | --- |
| **Event** |  |
| Alive | 84 |
| Dead | 92 |
| **Stage** |  |
| Ⅰ | 21 |
| Ⅱ | 145 |
| III | 3 |
| Ⅳ | 4 |
| X | 3 |
| **Grade** |  |
| G1 | 30 |
| G2 | 94 |
| G3 | 48 |
| G4 | 2 |
| GX | 2 |
| **Age** |  |
| ≤ 65 | 93 |
| ＞65 | 83 |
| **T Stage** |  |
| T1 | 7 |
| T2 | 24 |
| T3 | 140 |
| T4 | 3 |
| TX | 2 |
| **N Stage** |  |
| N0 | 49 |
| N1 | 122 |
| NX | 5 |
| **M Stage** |  |
| M0 | 79 |
| M1 | 4 |
| MX | 93 |
| **Gender** |  |
| Female | 80 |
| Male | 96 |
| **Alcohol** |  |
| NO | 64 |
| YES | 100 |
| Unknown | 12 |
| **Radiation_therapy** |  |
| NO | 101 |
| YES | 32 |
| Unknown | 43 |
| **Chemotherapy** |  |
| NO | 60 |
| YES | 116 |
